# 1012. Protective Effects of Maternal Influenza Vaccine during Pregnancy and Breastfeeding on Risk of Infant Influenza

**DOI:** 10.1093/ofid/ofad500.044

**Published:** 2023-11-27

**Authors:** Anne-Marie Rick, Helen D’Agostino, Hui Liu, John V Williams, G K Balasubramani, Judith M Martin

**Affiliations:** University of Pittsburgh, Pittsburgh, PA; University of Pittsburgh, Pittsburgh, PA; University of Pittsburgh, Pittsburgh, PA; University of Pittsburgh, Pittsburgh, PA; University of Pittsburgh, Pittsburgh, PA; University of Pittsburgh, Pittsburgh, PA

## Abstract

**Background:**

Children < 6 months of age are not eligible for influenza vaccine but are at high risk of influenza and its complications. Maternal influenza immunization (MII) in pregnancy or during lactation are two ways to provide infants vaccine specific influenza antibodies that could confer protection. Limited data delineate the individual and combined protective effects of MII and breastfeeding on influenza in infants. We aimed to evaluate the independent and combined protective effects of MII during pregnancy and breastfeeding against influenza during the first 6 months of life.

**Methods:**

We utilized a retrospective cohort of mother-infant pairs, including infants born between January 1, 2012, to December 31, 2019 with longitudinal care within a single health system. Maternal influenza vaccine, breastfeeding and important covariates (Table 1) were obtained from electronic health records. We evaluated the odds of laboratory-confirmed influenza among infants < 6 months of age based on MII in pregnancy and breastfeeding status at 3 months of age using unadjusted and adjusted logistic regression following stepwise regression for covariates with p-value < 0.15.

**Results:**

Of the 44,132 mother-infant pairs included, 0.3% (N=141) of infants had laboratory-confirmed influenza between birth and 6 months of age. 54% of infants received some/exclusive breastmilk at 3 months of age; 51% of mothers had MII during pregnancy. Breastfeeding alone (aOR: 0.77; 95%CI: 0.51-1.16) did not significantly protect against influenza compared to infants who received no breastmilk and no MII. In contrast, exposure to MII trended toward decreased risk of influenza by 37% (aOR: 0.63; 95%CI: 0.39-1.01), and the combination of MII in pregnancy and breastfeeding significantly decreased odds of infant influenza by 60% (aOR: 0.40; 95%CI: 0.25-0.66) (Table 1).

**Figure:**
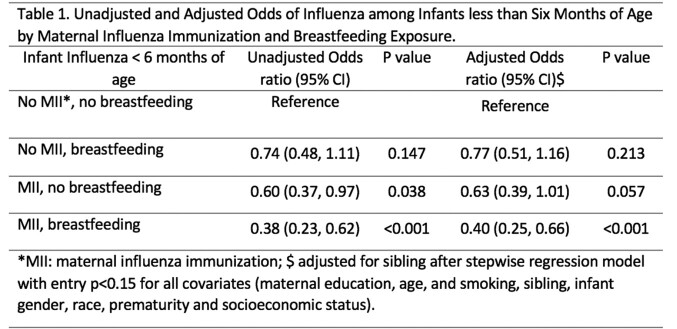

**Conclusion:**

MII in pregnancy can effectively reduce the risk of influenza between 40-60% in children < 6 months. Breastfeeding significantly enhances that protection, either through its broad immune properties and/or the vaccine-specific antibodies in milk. Increasing the administration and acceptance of MII in pregnancy and improving sustained breastfeeding rates may help reduce the burden of infant influenza.

**Disclosures:**

**John V. Williams, MD**, Merck: Grant/Research Support|Quidel: Board Member **Judith M. Martin, MD**, Merck, Sharp and Dhome: Grant/Research Support

